# Dermatoscopic yellow-white globules in primary balloon cells melanoma

**DOI:** 10.1016/j.jdcr.2025.03.012

**Published:** 2025-03-30

**Authors:** Emmanuel Ribereau-Gayon, Cédric Jalles, Olivier Harou, Anne-Laure Breton-Guitarian, Nelly Youssef-Provençal, Luc Thomas

**Affiliations:** a Department of Dermatology, Lyon Sud Hospital Center, Pierre-Bénite, France; b Claude Bernard University Lyon 1, Lyon, France; c Department of Dermatology, Macon Hospital Center, Macon, France; d Department of Anatomic Pathology, Lyon Sud Hospital Center, Pierre-Bénite, France; e Department of Dermatology, Saint-Joseph and Saint-Luc Hospital Centre, Lyon, France; f Cypath Dermpath Pathology Laboratory, Lyon, France; g Lyon Cancer Research Centre, Unité Mixte de Recherche de l'Inserm U1052 et du Centre National de la Recherche Scientifique 5286, Université Claude Bernard Lyon 1, Lyon, France

**Keywords:** dermatopathology, dermatoscopy, melanoma

## Introduction

Balloon cell neoplasms represent a very rare histopathological form of melanocytic lesion and their clinical and dermatoscopic features have not been well described. Histopathologically they are defined by a predominance of larges cells with small central basophilic nuclei and abundant clear and foamy cytoplasm.^1^ This appearance results from progressive vacuolization and merging of melanosome organelles. Consequently, the cells are less pigmented, giving a yellow-white coloration in dermatoscopy.^2^ Yellow-white globules have almost exclusively been described in balloon cell nevi (BCN), whereas most of dermatoscopic features of the rare reported cases of primary balloon cells melanoma (BCM) were yellow-white translucent areas. We highlight in this report the clinical, dermatoscopic and histopathologic features of 2 cases of primary BCM with prominent dermatoscopic yellow-white globules.

## Case description

### Patient 1

**Fig 1:**
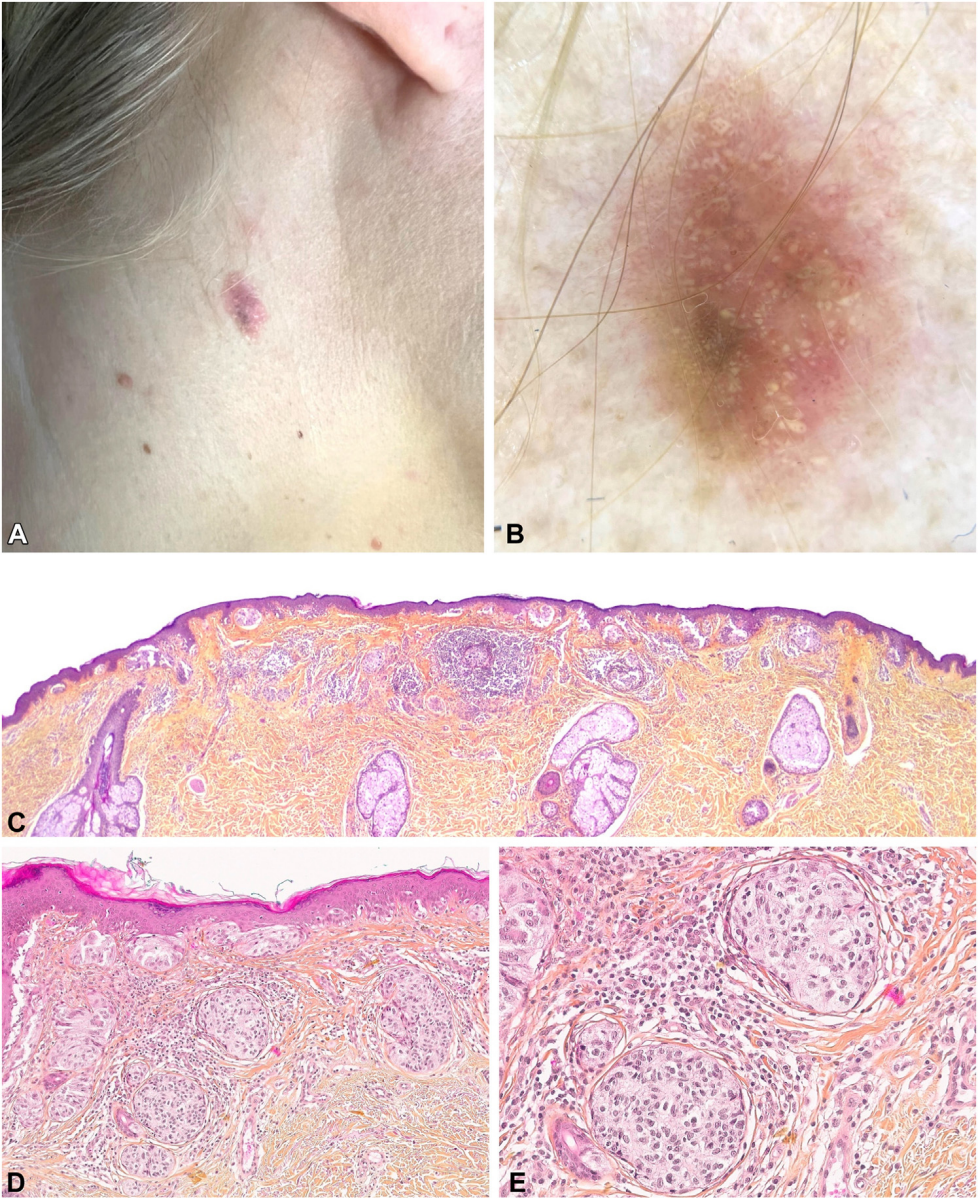
Patient 1: cervical unpigmented lesion (**A**) with prominent yellow-white globules, asymmetric brown network, dotted vessels, polychromia (**B**). Histopathologic examination showing a compound melanocytic lesion with a prominent population of atypical fusiform ballooning melanocytes in the dermis. **C,** low-power view (×25) showing the compound architecture of the lesion; **D,** medium-power view (×100) highlighting nests of atypical melanocytes; **E,** high-power view (×200) of the prominent population of atypical fusiform ballooning melanocytes.

A woman in her 30s, with no personal or family history, presented with an asymptomatic nodular lesion on the right side of her neck, which had appeared over a few months. Examination showed Fitzpatrick skin type II and a right cervical nodular, large, mostly unpigmented lesion (Fig 1). Dermatoscopy revealed a focal asymmetric homogeneous brown network associated with yellow-white globules of varying sizes, and asymmetric dot and globule vessels. Pathologic examination revealed a compound melanocytic lesion composed of nested melanocytes with some areas of intraepidermal pagetoid spread and a population of atypical fusiform ballooning melanocytes invading the dermis, representing >50% of the lesion. A diagnosis of primary BCM with a thickness of 1.1 mm, occurring within a preexisting nevus, was rendered.

### Patient 2

**Fig 2:**
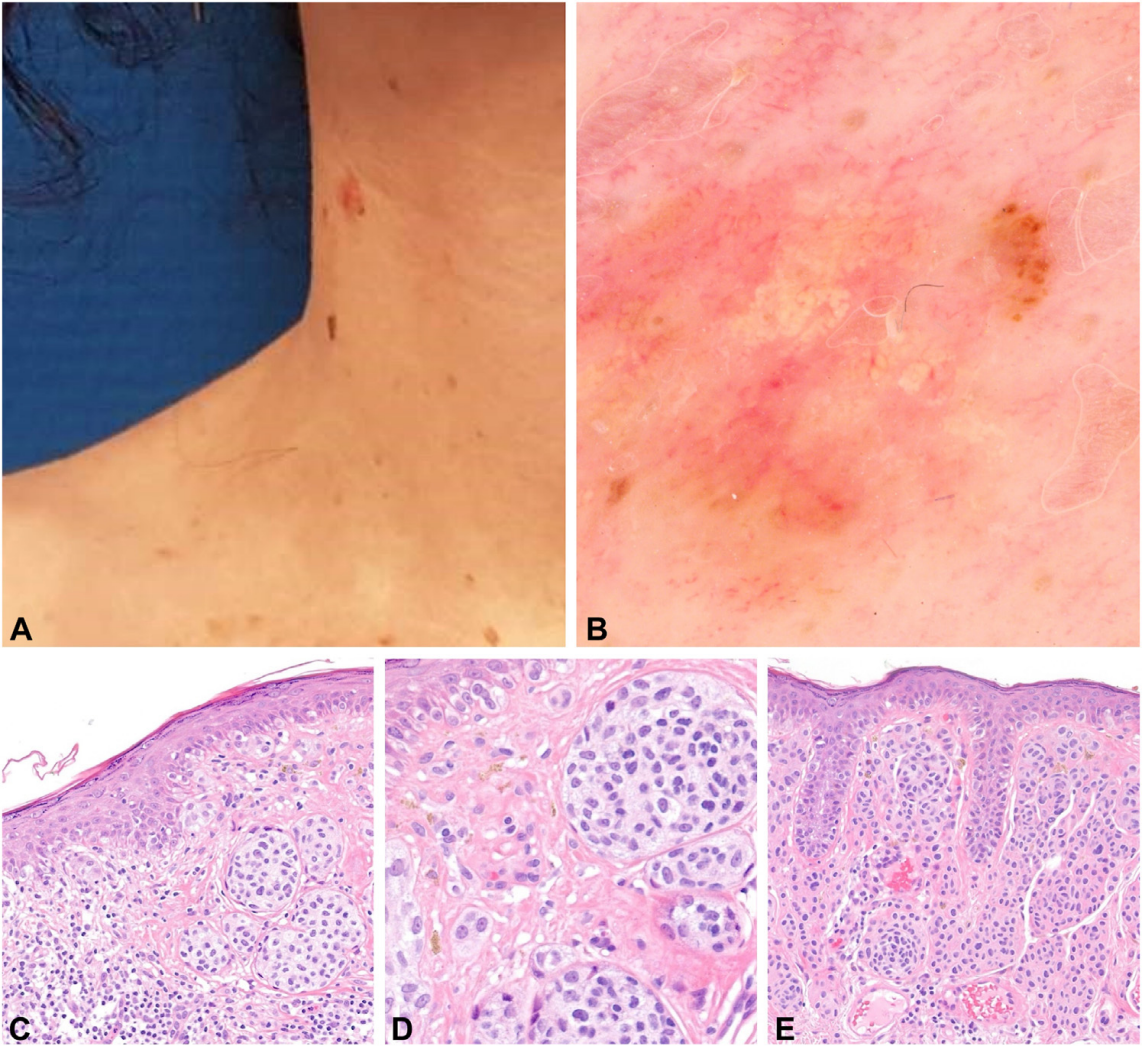
Patient 2: cervical unpigmented lesion (**A**) with central yellow-white globules, asymmetric brown globules, and polymorphous vessels (**B**). Histopathologic examination showing a compound melanocytic lesion with a population of mildly atypical ballooning melanocytes in the epidermis and the papillary dermis (**C, D**) associated with a population of melanocytes without atypia (**E**). (**C-E,** Hematoxylin-eosin stain; original magnifications: **C,** ×100; **D** and **E,** ×200.)

A woman in her 30s, without personal or family medical history, reported the recent modification of right cervical lesion with enlargement and itching. Clinical examination showed Fitzpatrick skin type IIIA and a right cervical nodular achromic lesion (Fig 2). Dermatoscopic examination showed a predominant achromic lesion with a focal asymmetric globular pattern, white globules, and polymorphous vessels (linear irregular, globular, and curved vessels). Pathologic examination showed 2 distinct populations of melanocytes: a compound proliferation of variable sized nests composed of mildly atypical ballooning melanocytes, associated with marked pagetoid spread, and a lateral dermal proliferation of small, monomorphous melanocytes without atypia. The balloon melanocytes composed >50% of the lesion. A diagnostic of primary BCM occurring from a preexisting nevus, with a thickness of 0.5 mm, was made.

## Discussion

Ballooning of melanocytes occur in both melanocytic nevi and melanomas.^1^ If small foci of ballooning melanocytes are common in nevi, BCN and BCM, which are defined by balloon cells representing >50% of the melanocytic cells, are rarely encountered lesions. Moreover, BCM represent one of the rarest histologic form of melanoma (0.15%).^1^

Although clinical appearance of balloon cell melanocytic neoplasms is not distinctive, some dermatoscopic features have been described. Yellow-whites globules have been reported in BCN, histopathologically correlated with balloon cell nevi nests.^2^^,^^3^ These lesions are reported mostly in young patients, over the head and neck, as pigmented and symmetric moles. In primary BCM, dermatoscopic features have been described only in 10 patients, with a predominant yellow-white structureless areas and rather than proper yellow-white globules.^4^, ^5^, ^6^, ^7^, ^8^ In 3 of these patients however, although yellow-white globules were described by the authors, they were not reproducing the typical features observed in BCN.^5^^,^^7^ Other dermatoscopic features described included hairpin, curved, and serpentine vessels (75%), yellow-white lines (50%), brown coloration (38%), and asymmetry (25%).^8^ These lesions occurred in older patients, over the extremities and the trunk, as large mole (often >1 cm), unpigmented and ulcerated in over half of the cases in a recent systematic review.^8^ Of note, 53% of the BCM described in this review were metastatic lesions of primary melanoma with ballooning cytology in only 20% of cases. In our cases, patients were young, and their primary BCM were located on the neck and the yellow-white globules were typical. In these recently changing lesions, association of other dermatoscopic criteria increased our suspicion index for melanoma, including focal and asymmetric reticular or globular pattern, architectural disorder, polychromia, and polymorphous vessels.

Dermatoscopic differentials for yellow-white globules include basal cell carcinomas (globules most often associated with arborizing telangiectasias), milia cysts (globules better visualized in nonpolarized light), and sebaceous hyperplasia (less well-defined globules with a “popcorn” appearance, a central crater, and coronal vessels).^9^

Histopathologically, in a prominent balloon cell melanocytic lesion, distinguishing between BCN and BCM might be challenging. The presence of greater nuclear pleomorphism, atypia, and higher Ki67 mitotic index, help distinguish BCM.^1^ However, >70% of BCM have been reported to have a component of conventional melanocytes.^1^ Average Breslow thickness of BCM has been evaluated at 2.6 mm, reflecting the palpable nature of the lesions.^8^ BCM is thus regarded as a vertical growth phase melanoma since there are no reported cases of in situ BCM or BCM with a junctional component of balloon cells. Of note, for comparable Breslow thickness, prognosis of BCM is not different from more classic histopathologic subtypes.^10^ Minor ballooning cell component (<50%) can be observed in several other melanomas subtypes, without particular significance.

In conclusion, our cases demonstrated that aside BCN, basal cell carcinoma, and sebaceous hyperplasia, yellow-white globules can be observed in primary BCM, and therefore cannot be considered a reassuring feature, especially when associated with dermatoscopic criteria for a melanocytic lesion.

## Conflicts of interest

Dr Thomas received equipment (dermatoscopy, photodermatoscopy, and digital dermatoscopy) provided to his institution by Casio, FotoFinder, HEINE, 3Gen, and C-Cube. The other authors have no conflicts of interest to declare.
